# Subtype-Specific Aptamer Technologies for Breast Cancer: From Molecular Recognition to Precision Applications

**DOI:** 10.3390/bios16100549

**Published:** 2026-10-01

**Authors:** Huibin Wu, Zhi Zeng, Wenyi Huang, Xinyan Lin, Rui Tian, Jianmin Chen, Qiulong Zhang

**Affiliations:** 1 Key Laboratory of Pharmaceutical Analysis and Medicine of Fujian Province, School of Pharmacy and Medical Technology, Putian University, Putian 351100, China; 13101430061@163.com (H.W.); zengzhi@ptu.edu.cn (Z.Z.); 15105942669@163.com (W.H.); nzy670@126.com (X.L.); 2 College of Biological and Food Engineering, Hubei MinZu University, Enshi 445000, China; 2021060@hbmzu.edu.cn

**Keywords:** breast cancer, aptasensor, liquid biopsy, theranostics

## Abstract

Breast-cancer management depends on reproducible molecular classification, yet tissue-based assays provide only spatially and temporally restricted views of a heterogeneous disease. Aptamers are programmable single-stranded DNA or RNA ligands that can integrate molecular recognition with biosensing, liquid-biopsy capture and targeted delivery. This review follows the translational chain from aptamer selection and molecular recognition to subtype resolution, signal or cargo conversion, biological validation, and clinical translation. We compare HER2-directed aptamers, ERα- and PHB2-associated tools for hormone-receptor-positive disease, and phenotype-oriented ligands for triple-negative breast cancer. We further organize aptasensors by signal-conversion mechanism, distinguish analytical performance in buffer from that in clinical matrices, and clarify the complementary role of aptamers in liquid-biopsy workflows, including CTC and extracellular-vesicle enrichment alongside separate ctDNA sequence-variant analysis. For theranostic systems, affinity alone is insufficient; pharmacokinetics, receptor trafficking, endosomal escape and comparator-controlled efficacy ultimately determine functional benefit. Aptamers are therefore most compelling as modular recognition elements for multiplexing, reversible engineering, and targeted delivery. Progress will depend on transparent selection reporting, orthogonal target validation, and prospective multicenter studies.

## 1. Introduction: The Clinical Problem That Aptamer Technologies Must Solve

### 1.1. Epidemiologic and Molecular Context

Breast cancer remains one of the most frequently diagnosed malignancies worldwide, with approximately 2.3 million new cases and 685,000 deaths estimated in 2020 [1,2]. Earlier detection and increasingly effective systemic therapies have improved outcomes, but prognosis still varies sharply with stage, tumor biology, and treatment response [1,3]. This heterogeneity makes breast cancer an archetypal test of whether a molecular-recognition technology can progress from analytical performance to clinically useful stratification.

Gene-expression studies revealed intrinsic molecular patterns, including luminal-like, HER2-enriched and basal-like subtypes, which were later refined into clinically relevant categories such as luminal A and luminal B, whereas routine care uses clinicopathological surrogates based principally on estrogen receptor (ER), progesterone receptor (PR), HER2, and proliferation [4,5]. These frameworks overlap but are not interchangeable. A HER2-positive clinicopathological tumor is not necessarily classified as HER2-enriched by PAM50, and triple-negative disease is not synonymous with the basal-like intrinsic subtype. The distinction matters because many aptamer studies assign a “subtype” from one cell line or a limited receptor panel. Such models are valuable for discovery, but they do not reproduce the diversity of a clinical category.

Current classification remains anchored in tissue biopsy followed by immunohistochemistry (IHC) and, when indicated, in situ hybridization (ISH). These assays are clinically validated and cannot be displaced by analytical sensitivity alone. Nevertheless, a tissue sample is a static and spatially restricted view of a disease that evolves under treatment. Sampling bias, intratumoral heterogeneity, and receptor conversion between primary and metastatic sites can all uncouple a historical tissue result from the tumor population present at a later decision point [3,5]. Programmable recognition elements could add value if they enable repeated, multiplexed or spatially resolved measurements that complement—not merely replicate—standard pathology.

### 1.2. Five Unmet Needs as Translational Benchmarks

Five needs provide a practical benchmark for the technologies reviewed here. First, HER2 testing must reproducibly distinguish IHC 0, 1+, 2+/ISH-negative and amplified or overexpressed disease when those distinctions determine eligibility for an antibody–drug conjugate. The 2023 ASCO–CAP update retained established HER2 interpretation while emphasizing the clinical relevance of accurately discriminating low levels of membrane staining [6]. Consequently, an aptamer assay should not be described as solving HER2-equivocal or HER2-low classification unless it is compared with a validated tissue reference method and linked to the intended treatment context.

Second, neoadjuvant, adjuvant, and metastatic therapy create a need for longitudinal molecular monitoring without serial invasive biopsies. Third, treatment of triple-negative breast cancer (TNBC) increasingly depends on heterogeneous and sometimes dynamic targets such as Trop-2, HER2, PD-L1, and other surface phenotypes; bulk positivity can conceal clinically relevant subpopulations. Fourth, epithelial-marker-dependent circulating tumor cell (CTC) platforms can miss cells that have downregulated EpCAM during epithelial-to-mesenchymal transition. Fifth, decentralized testing requires low-cost and operationally robust formats whose performance is demonstrated in whole blood, plasma, or serum—not only in optimized buffer.

**Figure 1:**
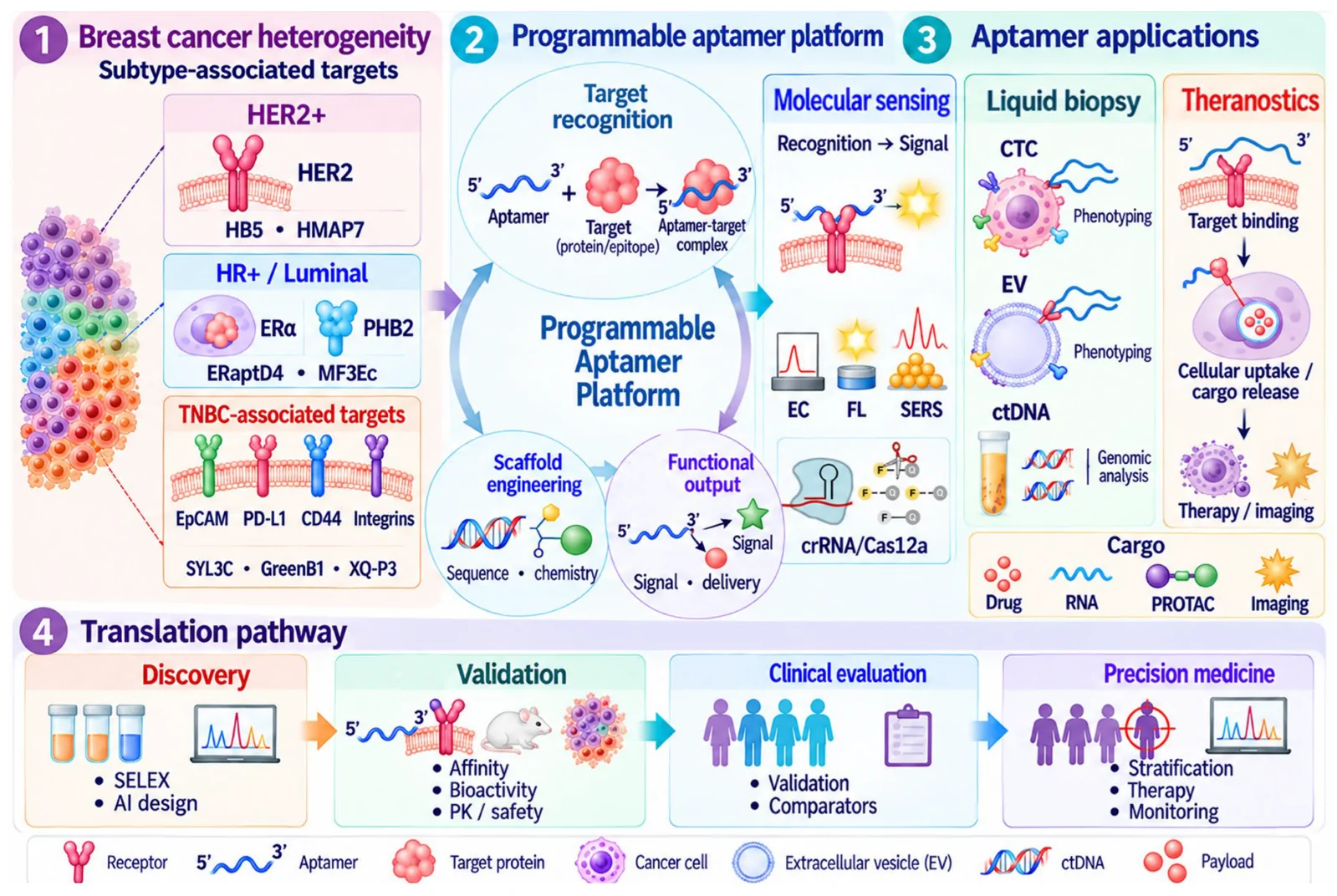
Overview of the programmable aptamer framework for breast cancer precision applications. (**1**) Breast cancer molecular heterogeneity and representative molecular targets. (**2**) Programmable aptamer platform integrating target recognition, scaffold engineering, and functional output. (**3**) Aptamer applications in molecular sensing, liquid biopsy, and theranostics. (**4**) Translational continuum from discovery and validation to translation and precision medicine.

These benchmarks deliberately separate analytical validity from clinical utility. A femtogram-per-milliliter limit of detection (LOD) may be technically impressive, but it does not establish that a test improves classification, predicts benefit or changes management. The field will ultimately be judged by clinically meaningful performance against a prespecified comparator. These breast-cancer-specific needs define the biological and translational context in which aptamer technologies should be evaluated (Figure 1).

### 1.3. Why Aptamers—And Where the Comparison with Antibodies Stops

Aptamers are short single-stranded oligonucleotides that adopt defined secondary and tertiary structures. Shape complementarity, electrostatic contacts, hydrogen bonding, base stacking, and other non-covalent interactions can support affinities from the micromolar to picomolar range, depending on the target and measurement context [7,8,9]. SELEX and its derivatives iteratively enrich binding sequences from large combinatorial libraries, while cell-SELEX preserves native membrane presentation and can select for a phenotype without a predefined molecular target [10,11,12,13].

Their principal advantage is programmability. Solid-phase synthesis enables defined placement of fluorophores, redox reporters, linkers, modified nucleotides, and therapeutic cargos; complementary strands permit reversible assembly; and multiple aptamers can be arranged on nanostructures at controlled valency and spacing. These properties can reduce some sources of biological batch variability and simplify integration with electrochemical, optical, and nucleic-acid amplification systems [7,9,14]. Pegaptanib established that an aptamer can reach regulatory approval when target biology, local administration, and exposure are aligned, but this precedent does not by itself validate systemic oncology applications [15].

Work on aptamers against receptor tyrosine kinases provides a broader mechanistic context for breast-cancer applications and underscores how receptor conformation and trafficking influence function [16]. In breast cancer, HER2 membrane presentation, intracellular Erα, and heterogeneous TNBC phenotypes impose different requirements on molecular recognition [3,5,6,17]. These differences determine whether an aptamer is useful for tissue staining, cell or vesicle capture, or targeted delivery [9,17,18]. Programmable attachment and multiplex assembly are potential advantages, but their value depends on preserving recognition under the conditions required by the intended application [9].

Comparisons with antibodies require qualification. Antibodies benefit from mature manufacturing, long systemic half-lives, bivalent avidity, and extensive clinical validation. Aptamers are smaller and more readily synthesized and modified, but unmodified sequences are rapidly cleared, surface immobilization can disrupt folding, and target engagement can be highly dependent on ionic conditions. Claims of universally lower cost or absent immunogenicity also overlook modification, formulation, quality-control, and complement-related risks. Aptamers should therefore be developed as complementary recognition modules in applications where programmability, multiplexing, or reversible assembly produces a demonstrable functional advantage.

### 1.4. Scope and Organization

This review is organized around the chain from molecular selection to clinical translation. We first examine how selection context and chemical optimization shape function; then compare subtype-oriented aptamers, signal-conversion architectures, liquid-biopsy applications, and theranostic constructs. We close with a staged roadmap in which target identity, matrix performance, pharmacology, and clinical comparison are treated as translational gates. Since the introduction of in vitro selection and SELEX in 1990 and later cell-based selection, breast-cancer aptamer research has expanded from single-target binding to subtype discrimination, biomarker discovery, and integrated diagnostic–therapeutic systems [11,12,13]. The central question is no longer whether an aptamer can bind, but whether programmable recognition remains informative after biological and clinical constraints are imposed. The overall conceptual framework of this review is illustrated in Figure 1, which highlights how programmable aptamer recognition can connect breast cancer molecular heterogeneity with sensing, liquid biopsy, and therapeutic applications.

Previous reviews have primarily addressed aptamer selection, sensing, diagnosis, and therapy in breast cancer [19,20,21]. This review instead integrates subtype-associated target biology with intended use while distinguishing analytical performance, patient-level evidence, and clinical utility (Table 1).

### 1.5. Review Methodology

To improve the transparency and reproducibility of this narrative review, a structured literature search was conducted during manuscript revision. PubMed was used as the principal bibliographic database, supplemented by X-MOL Advanced Search for additional literature discovery. Searches were performed on 17 September 2026, covering publications from 1990 to 2026. The PubMed core search combined breast cancer-related terms with aptamer-related terms, including aptamer, aptasensor, SELEX, and systematic evolution of ligands by exponential enrichment. Additional targeted searches addressed molecular subtypes, biosensing, liquid biopsy, and theranostic applications. Detailed database-specific search strategies, field restrictions, date limits, and retrieval counts are provided in Supplementary Table S1.

The PubMed searches retrieved 961 records in the core search, 273 in the molecular-subtype search, 381 in the biosensing search, 142 in the liquid-biopsy search, and 550 in the theranostic search. Because these search sets overlapped substantially, the counts were not summed and should not be interpreted as the number of unique or included studies. X-MOL searches were used as a supplementary discovery step, and bibliographic details of potentially relevant records were verified against the corresponding primary publications before citation.

Primary studies were prioritized when they reported breast-cancer-relevant aptamer selection, molecular recognition, target validation, biosensing, liquid-biopsy applications, imaging, targeted delivery, or therapeutic evaluation. Relevant review and methodological articles were additionally used to provide context for aptamer selection, analytical validation, pharmacology, and translational considerations. Duplicate records, conference abstracts lacking sufficient methodological information, and studies without direct relevance to breast cancer or to the mechanistic and translational scope of this review were excluded. Reference lists and citing articles of key publications were additionally examined to identify relevant studies not captured by the structured searches.

## 2. From Sequence Selection to Functional Recognition

### 2.1. Choosing a Selection Strategy That Matches the Intended Use

The selection target is the first determinant of translational behavior. Protein-SELEX is appropriate when a target is known, purifiable, and presented in a biologically relevant state. It simplifies counter-selection and target attribution, but immobilization, tags, and absence of native glycosylation can expose artificial epitopes. Peptide-SELEX magnifies this risk: a ligand may bind the isolated sequence strongly while recognizing the folded extracellular domain or membrane receptor much more weakly. HB5 illustrates the problem. The aptamer was selected against a HER2 peptide and was reported to bind that peptide at approximately 18.9 nM but the extracellular domain at approximately 316 nM [18]. These values describe different analytes and should not be merged into a single “HER2 affinity.”

Cell-SELEX is preferable when native conformation, post-translational modification, receptor density, or an incompletely defined phenotype is central to the intended application. Positive selection on MCF-7, BT-474, SK-BR-3 or MDA-MB-231 cells, combined with counter-selection against other breast-cancer or non-malignant mammary lines, can enrich subtype-biased binders [10,22,23,24]. The gain in biological context is offset by target uncertainty and sensitivity to culture conditions. A sequence may recognize a glycoform, protein complex, or culture-specific surface state rather than a stable tumor marker.

Differential designs sharpen the biological question. Loss–gain cell-SELEX can alternate target-positive and target-deficient cells to select a ligand associated with a defined molecule, as used in the selection of the PD-L1 aptamer XQ-P3 [25]. Exo-SELEX selects against intact extracellular vesicles and can reveal vesicle-associated phenotypes that purified-marker selection would miss [26]. High-throughput sequencing (HTS) adds temporal resolution by tracking millions of sequences across rounds. Ferreira and colleagues used cell-SELEX with HTS to identify Apt1 and Apt2 against MDA-MB-231 cells, with reported cell-binding dissociation constants of 44.3 ± 13.3 nM and 17.7 ± 2.7 nM, respectively [23]. However, read abundance is influenced by amplification and sequencing bias; enrichment is not an affinity measurement.

**Figure 2:**
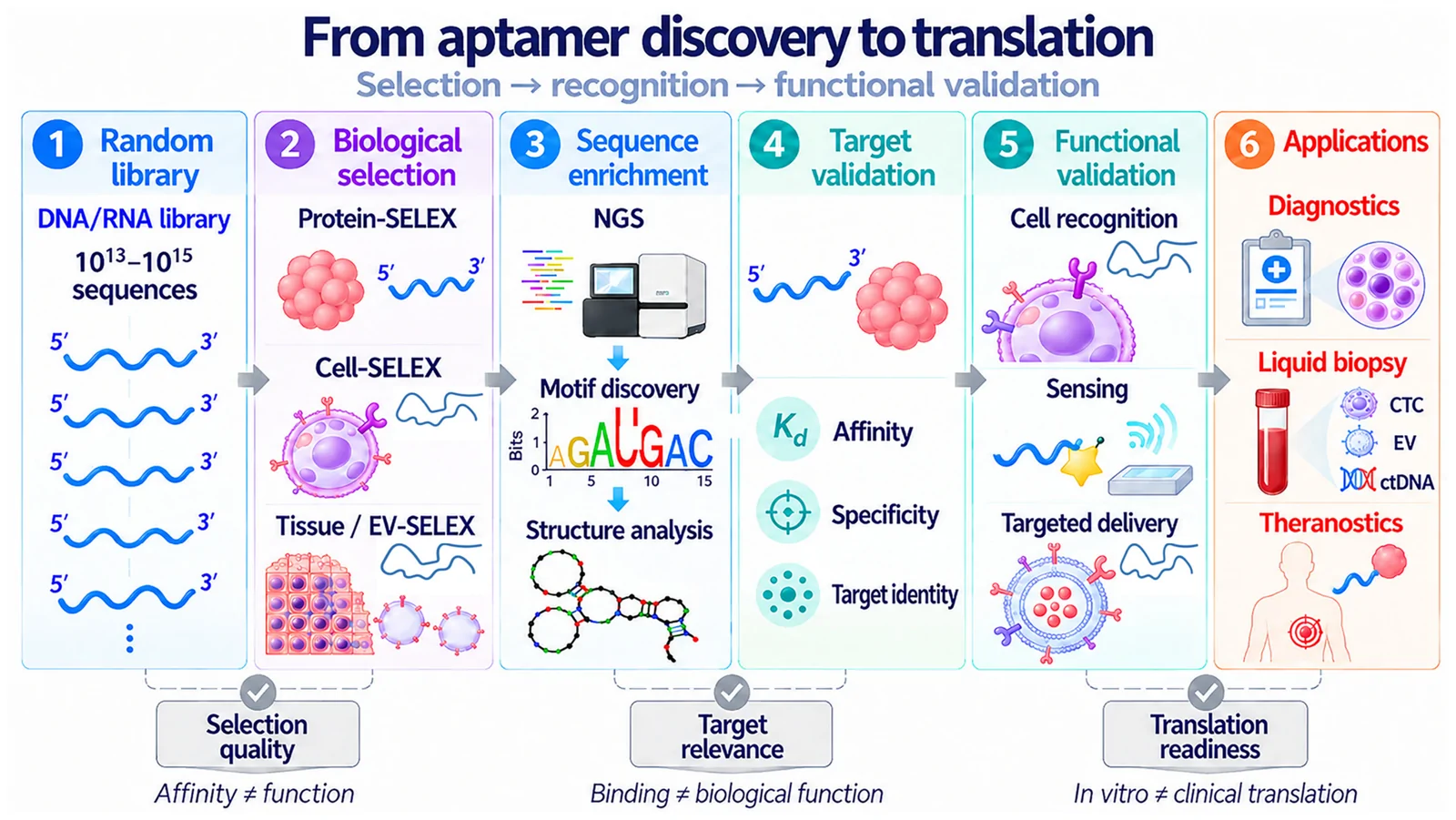
Aptamer discovery and translational pipeline. (**1**) Random DNA/RNA library generation. (**2**) Biological selection using different SELEX strategies. (**3**) Sequence enrichment and bioinformatic analysis. (**4**) Molecular target validation. (**5**) Functional validation in sensing and therapy. (**6**) Translational applications in diagnostics, liquid biopsy, and therapeutics.

Accordingly, selection strategy should follow the use case. A soluble-protein sensor needs reproducible recognition of the clinically measured analyte. A delivery ligand requires target accessibility, receptor-mediated internalization, and productive trafficking in living cells. A subtype-discrimination probe requires counter-selection that represents the clinical alternatives. No SELEX format is intrinsically superior outside that context. Selection and downstream validation should therefore be considered as a continuous pipeline from ligand enrichment to functional and translational evaluation (Figure 2).

### 2.2. Selection Pressure, Bias, and Minimum Reporting

SELEX conditions determine the phenotype of the recovered ligand. Ionic composition affects nucleic-acid folding; target concentration, incubation time, and wash stringency influence selection outcomes; target immobilization may alter epitope accessibility; and PCR bias can enrich sequences with superior amplification efficiency rather than binding performance [27,28]. Therefore, describing a protocol simply as “8–20 rounds of increasingly stringent selection” is insufficient for evaluating aptamer reproducibility.

Minimum reporting should include library composition, target presentation, buffer conditions, selection parameters, counter-selection strategy, amplification details, sequencing information, and the complete validated sequence [27,28]. For breast-cancer applications, authenticated cell lines, receptor-expression profiles, and rationally designed counter-selection models are essential for supporting subtype-specific recognition claims.

### 2.3. Target Deconvolution as a Translational Gate

Cell-selected aptamers can function as discovery reagents, but target deconvolution is not an optional mechanistic exercise. It determines whether expression can be measured in tumors and normal tissues, whether a therapeutic ligand is likely to internalize, and whether off-tumor binding is predictable. Common approaches include aptamer-mediated pull-down followed by mass spectrometry, photo-crosslinking, competition with antibodies or ligands, gene knockdown or knockout, and rescue experiments [29].

Three breast-cancer examples show both the value and the limits of the approach. MF3Ec was derived from an MCF-7-selected aptamer and later associated with plasma-membrane prohibitin 2 (PHB2), nominating a noncanonical surface form of PHB2 as a potential luminal A marker [24,30]. PDGC21T was linked to integrin α3 (CD49c) through aptamer-mediated immunoprecipitation and LC–MS/MS [31]. GreenB1 was identified as an internalizing ligand for TNBC cells and shown to bind β1-integrin using orthogonal biochemical assays [32]. These findings are mechanistically informative, but they do not establish universal subtype specificity. Translation requires quantitative tissue distribution, assessment across independent tumor cohorts, and confirmation that loss of the candidate target abolishes binding.

### 2.4. Chemical and Structural Optimization

Unmodified RNA and many short DNA aptamers are vulnerable to nucleases and renal filtration. Common responses include 2′-fluoro-pyrimidine and 2′-O-methyl substitutions, locked nucleic acids, phosphorothioate linkages, terminal caps, PEG or lipid conjugation, and incorporation into nanoparticles [7,8,9]. Multivalent DNA tetrahedra, three-way junctions, and origami-like scaffolds can increase apparent binding through avidity and improve transducer geometry [14]. These interventions should be viewed as a multivariable optimization rather than a linear stability upgrade.

Modification can alter folding, hydration, protein interactions, and target affinity. Large conjugates can extend circulation but reduce tumor penetration or introduce formulation-specific toxicity. Post-selection truncation may remove amplification handles and nonessential regions, lower synthesis cost, and sometimes improve affinity, as reported when the 95-nt MF3 parent was shortened to 53-nt MF3Ec [24]. However, every modified or truncated product is a new molecular entity whose binding and biological function must be remeasured under intended conditions.

### 2.5. From Enrichment to Functional Validation

A four-stage validation framework can bridge the gap between sequence enrichment and translational function. Affinity should be confirmed by quantitative and, when possible, orthogonal methods, while specificity requires competition assays, target-deficient models, and relevant counter-target controls [27]. Functional validation should demonstrate the intended biological output under relevant conditions, and therapeutic candidates require additional evaluation of stability, pharmacokinetics, biodistribution, and safety [8,9].

Affinity alone does not guarantee biological function: a low dissociation constant may not ensure accessible epitopes, efficient internalization, or intracellular delivery [9]. Conversely, moderate affinity can be sufficient when multivalent binding, high target abundance, or signal amplification contributes to performance.

## 3. A Subtype-Oriented Aptamer Toolbox

**Figure 3:**
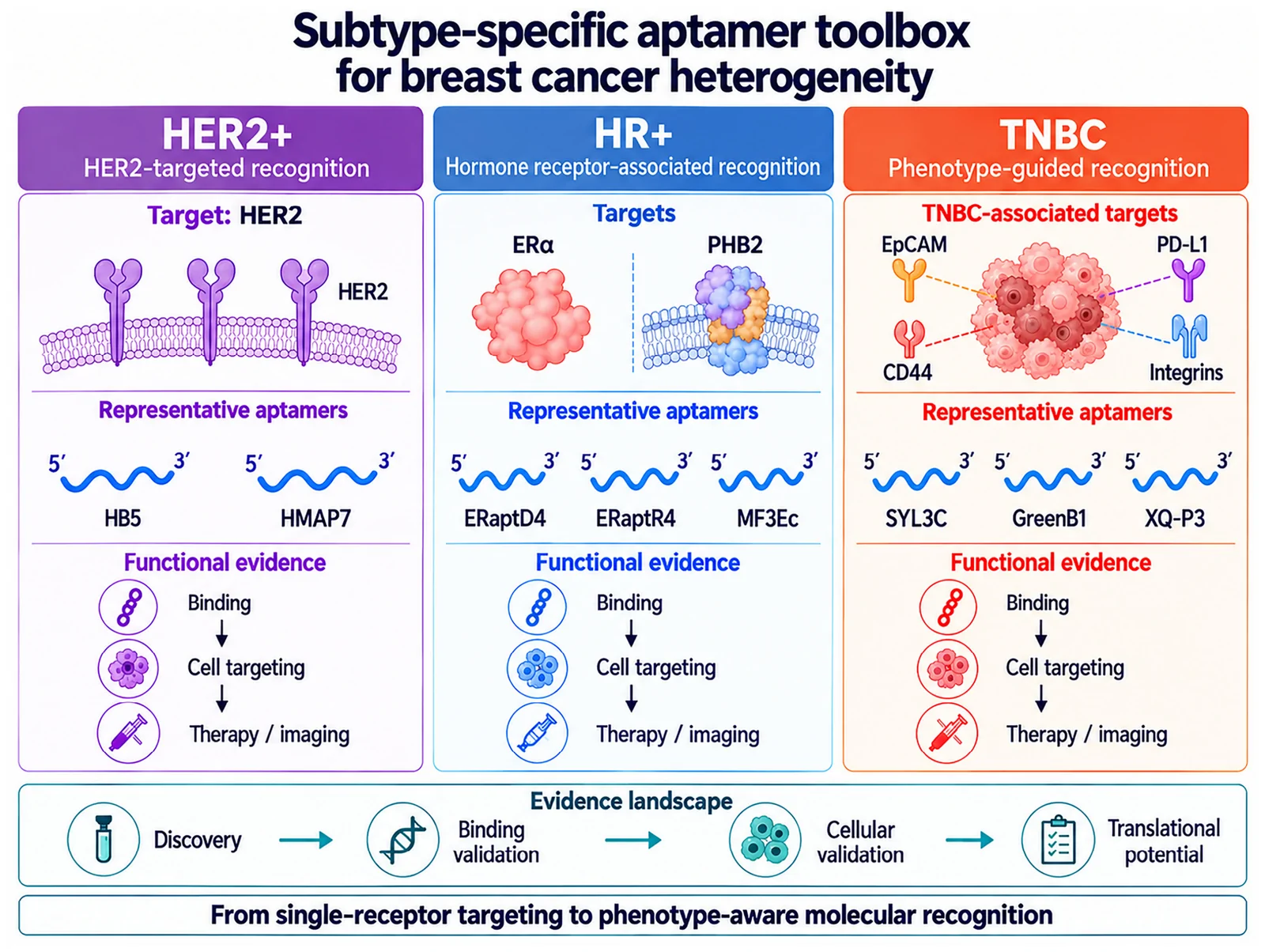
Subtype-specific aptamer toolbox across breast cancer heterogeneity. Representative targets, aptamers, and functional evidence are summarized for HER2-positive, HR-positive, and triple-negative breast cancer (TNBC), together with the progression from aptamer discovery and binding validation to cellular validation and translational potential.

Representative aptamers associated with HER2-positive, hormone-receptor-positive, and triple-negative breast cancer are organized as a subtype-oriented molecular toolbox in Figure 3.

### 3.1. HER2-Positive and HER2-Low Disease

#### 3.1.1. Target Biology and the Measurement Problem

HER2 is a receptor tyrosine kinase whose amplification or overexpression drives signaling through PI3K–AKT and MAPK pathways and defines a clinically actionable group of breast cancers [6]. IHC 3+ or IHC 2+/ISH-amplified tumors are eligible for established HER2-directed regimens, whereas lower, non-amplified expression has become relevant for trastuzumab deruxtecan-based treatment strategies [6,33]. “HER2-low” should be treated as a treatment-eligibility descriptor rather than a proven new intrinsic subtype [33]. This distinction raises the analytical bar: an aptamer sensor must quantify the relevant form of HER2 in the relevant specimen and demonstrate concordance with clinical assays; detecting recombinant HER2-ECD in buffer is not equivalent to classifying tissue membrane staining.

#### 3.1.2. HB5: A Widely Reused Ligand with Context-Dependent Affinity

The 86-nt DNA aptamer HB5 was selected against a HER2 peptide and subsequently applied in HER2-positive cell recognition, biosensing, drug delivery, and receptor-targeting studies [18,34,35,36,37,38,39,40]. The original study reported dissociation constants of approximately 18.9 nM for the peptide and 316 nM for the full extracellular domain (ECD) [18]. The approximately 17-fold difference highlights that affinity measured against an isolated epitope may not fully reflect recognition of the native receptor context. Nevertheless, HB5 can discriminate HER2-expressing cell models from negative controls and remains one of the most widely reused HER2 aptamer scaffolds.

The broad application of HB5 has also revealed the distinction between aptamer affinity and assay performance. HER2 aptamer-based electrochemical platforms incorporating enzymes, covalent organic frameworks, tetrahedral DNA structures, transition-metal composites, ZnO nanohybrids, nickel foams, and gold-modified electrodes have achieved increasingly low detection limits [35,36,37,38,39]. These improvements mainly originate from signal amplification and transducer engineering rather than enhanced intrinsic affinity of HB5 [19,27]. A fluorescence assay using HB5 for serum HER2-ECD detection demonstrated analytical feasibility [41]; however, available evidence remains insufficient to replace guideline-recommended HER2 testing approaches [6]. In addition, HER2-directed aptamer systems and lysosome-targeting nanoconstructs have demonstrated receptor degradation effects in breast cancer cells [40,42], supporting the potential of aptamer-mediated HER2 regulation.

#### 3.1.3. Cell- and ECD-Selected Alternatives

Cell-selected HER2-associated aptamers provide a useful contrast. HMAP7 and other recently reported HER2-directed aptamers showed preferential binding and internalization in HER2-overexpressing breast cancer cells, supporting theranostic development [43]. HMAP7 is a 41-mer with reported apparent Kd values of 260 ± 40 nM in MDA-MB-453 cells and 350 ± 70 nM in SK-BR-3 cells [43]; these values are weaker than some previously cited low-nanomolar estimates. The source studies do not establish a head-to-head ranking against HB5 under identical conditions, and target deconvolution or epitope definition remains incomplete. The same distinction is evident for the SK-BR-3-selected aptamer series: full-length sk6 showed the reported subnanomolar cell-binding affinity (Kd = 0.61 ± 0.14 nM), whereas the optimized 53-nt derivative sk6Ea exhibited weaker binding affinity (Kd = 49.32 ± 14.53 nM) [22]. Incomplete target definition limits mechanistic interpretation despite in vivo imaging in xenograft models [22].

An earlier RNA aptamer selected against ErbB2/HER2 extracellular domain promoted receptor degradation and inhibited tumorigenic growth in experimental models [40]. Together with a three-in-one aptamer–siRNA chimera targeting the EGFR/HER2/HER3 network [44], these studies demonstrate that receptor recognition can be coupled to functional perturbation. However, these approaches have not yet been benchmarked against clinically validated HER2 ADCs such as T-DM1 or trastuzumab deruxtecan [33,45].

#### 3.1.4. Design Inference and Unresolved Questions

Comparison across these ligands supports a cautious design inference: selection against soluble peptides or proteins may be sufficient for soluble-analyte sensing, whereas cell or native-domain selection may be more appropriate when internalization and receptor trafficking are required [27,28]. This is not an absolute rule—cell selection can yield unidentified or context-dependent targets, whereas protein selection can produce functional ligands [29].

Three gaps currently limit clinical interpretation. First, no high-resolution structure has defined a HER2–aptamer interface, limiting rational optimization and epitope comparison [7,27]. Second, prospective studies against validated HER2 reference assays remain lacking, particularly at IHC 0/1+ and 2+/ISH-negative boundaries [6]. Third, therapeutic constructs have not been rigorously compared with T-DM1 or T-DXd regarding exposure, efficacy, and toxicity [33,45]. Therefore, HER2 aptamers remain investigational recognition modules rather than validated companion diagnostics or therapeutic alternatives. Nevertheless, their programmable chemistry and compatibility with sensing, imaging, and targeted delivery provide a strong basis for further development once native-receptor recognition and clinical concordance are established [46].

### 3.2. Hormone-Receptor-Positive Disease

#### 3.2.1. Clinical Context

Hormone-receptor-positive cancers account for the largest clinicopathological group and encompass biologically diverse diseases. ER and PR IHC remains the validated basis for predicting endocrine sensitivity; gene-expression assays can refine selected adjuvant decisions, whereas no aptamer-based assay has entered routine receptor assessment [3]. The relevant opportunity is therefore complementary: standardized molecular detection, research imaging, or longitudinal measurement of phenotypes associated with luminal disease.

#### 3.2.2. ERα-Binding Aptamers

ERaptD4 is a 76-nt DNA aptamer reported to bind ERα with an association constant of (1.55 ± 0.298) × 10^8^ M^−1^ and to detect altered ERα expression using ELISA, immunoblotting, immunocytochemistry, and experimental tissue staining [17]. An association constant must not be presented as a dissociation constant; the reciprocal of the reported point estimate corresponds to approximately 6.45 nM under the same binding model and is not a separately measured Kd [17]. The study provides encouraging cross-assay evidence, but the limited tissue cohort supports further validation rather than replacement of IHC [17].

ERaptR4, a 17-nt RNA sequence, was identified through an in silico strategy based on estrogen-response-element-like motifs and was reported to bind ERα with an association constant of 1.02 × 10^8^ M^−1^ [47]. Docking suggested a possible interaction with the DNA-binding region, whereas proposed ERaptD4 interactions with the AF-1 region are likewise model-dependent. Neither binding interface has been structurally resolved. Electrochemical ERα aptasensors and amplification-assisted formats demonstrate that ER recognition can be translated into analytical signals [48,49], while ER-positive-cell aptamers and multicolor classification systems extend the concept to cellular phenotypes [50,51].

#### 3.2.3. MF3Ec and Membrane-Associated PHB2

MF3Ec provides a different route to luminal discrimination. The 53-nt truncated DNA aptamer was derived from cell-SELEX against MCF-7 cells and reported to discriminate this luminal A model from selected non-luminal breast-cancer models, with a cell-binding dissociation constant of approximately 18.95 nM [24]. A subsequent target-deconvolution study associated MF3Ec binding with membrane-associated PHB2 [30]. In a separate aptamer-histochemistry study of 140 formalin-fixed, paraffin-embedded tissue specimens (110 breast-cancer specimens and 30 controls), 24 of 30 luminal A cases were MF3Ec-positive; however, overlap with luminal B/HER2-negative disease limited stand-alone subtype discrimination [52]. The finding is notable because PHB2 is classically intracellular, whereas a reported tumor-associated membrane pool may provide a surface-accessible recognition site.

Mechanistic and translational claims nevertheless remain distinct. Colocalization and trafficking observations support an association between MF3Ec and PHB2 biology but do not define a high-resolution binding interface. Tissue staining in a limited cohort suggests subtype discrimination, whereas the prevalence, density, and normal-tissue distribution of membrane PHB2 require independent validation. Claims of luminal specificity should therefore remain restricted to the tested models and cohorts.

CA15-3/MUC1-directed platforms broaden luminal-associated detection but are not subtype-specific. Electrochemical aptasensors illustrate sensitive CA15-3 measurement [53,54], while broader analyses emphasize the limited subtype specificity of the marker itself [55]. Multiplexing ER, PR, HER2, and MUC1-related signals may improve phenotyping, provided that each measurement and the combined classification rule are independently validated.

#### 3.2.4. Translational Questions

Luminal aptamers face three priority questions. Is membrane PHB2 consistently accessible across clinically defined luminal A tumors and absent from relevant normal tissues? Can ERaptD4 add reproducible information to standardized ER IHC across laboratories and pre-analytical conditions? Can a ligand report receptor activity rather than abundance? The last possibility is conceptually attractive, especially for endocrine resistance, although no aptamer-based functional ER activity assay has yet been clinically validated. Combining ER-directed recognition with membrane-accessible luminal-associated targets may nevertheless provide complementary routes for molecular phenotyping beyond receptor abundance alone.

### 3.3. TNBC as a Phenotype Panel Rather than a Single Target

#### 3.3.1. Heterogeneity as the Organizing Principle

TNBC is defined clinically by the absence of ER and PR expression and lack of HER2 overexpression or amplification, rather than by a single shared surface antigen. Gene-expression analyses identified basal-like, mesenchymal, immune-associated, and luminal-androgen-receptor patterns, and later refinement showed that some original “immunomodulatory” and “mesenchymal stem-like” signals reflected infiltrating immune or stromal cells rather than tumor-intrinsic categories [56,57]. These classifications are research frameworks rather than routine companion diagnostics. Their key implication for aptamers is nevertheless robust: no single ligand can be assumed to capture all TNBC cells.

#### 3.3.2. Epithelial and ADC-Relevant Phenotypes

SYL3C is a 48-nt DNA aptamer selected against recombinant EpCAM and used in cell imaging and CTC capture [58]. Reported binding to MDA-MB-231 cells should therefore be interpreted cautiously because EpCAM expression can vary among TNBC models. The ligand has been integrated into a liquid-crystal platform for label-free single-cell detection [59], a dual-mode EpCAM biosensor [60], and an NIR-II SYL3C–PEG–ICG probe for image-guided resection in a mouse model [61]. EpCAM-directed aptamer–siRNA chimeras also enabled targeted gene knockdown in EpCAM-positive breast-cancer models [62]. These applications demonstrate modularity, but their target population is EpCAM-positive disease rather than TNBC as a whole.

Trop-2 is clinically relevant because the Trop-2-directed ADC sacituzumab govitecan improves outcomes in metastatic TNBC [63]. Aptamer-based colorimetric, electrochemical, and isothermal-amplification platforms have measured Trop-2 on breast-cancer cells [64,65,66]. Their analytical sophistication should not be conflated with companion-diagnostic validation. The studies use different aptamer constructs, target presentations, units and model matrices, and none establishes a threshold that predicts clinical benefit from ADC therapy [63].

#### 3.3.3. Mesenchymal and Invasive Phenotypes

Integrin-directed aptamers address phenotypes that may escape EpCAM-only capture. PDGC21T binds TNBC cells and was associated with CD49c/integrin α3 through immunoprecipitation and LC–MS/MS [31]. GreenB1 internalizes into MDA-MB-231 and MDA-MB-436 cells and binds β1-integrin under the reported experimental conditions [32]. These ligands are useful candidates for imaging and delivery, but two uncertainties remain: expression of integrin subunits on normal mammary and other tissues creates potential on-target/off-tumor binding, and no comparator-controlled therapeutic study has established an advantage over standard treatment.

An RNA aptamer that identified plasma-membrane ATP synthase beta subunit in aggressive cancer further illustrates the biomarker-discovery potential of phenotype selection [67]. The result should be treated as an early-marker hypothesis that requires independent tissue validation rather than a universal TNBC marker.

#### 3.3.4. Immune Phenotypes

The PD-L1 aptamer XQ-P3 was selected by loss–gain cell-SELEX and conjugated to paclitaxel. In reported TNBC models, the construct combined preferential delivery to PD-L1-high cells with competitive interference in the PD-1/PD-L1 axis [25]. This dual recognition-and-modulation design is attractive, but its predictive value, pharmacokinetics, and toxicity have not been defined in patients. Gint4.T, a PDGFRβ aptamer, potentiated anti-PD-L1 therapy in preclinical TNBC models by modulating tumor-cell signaling and immune-cell infiltration [68]. These findings support combination strategies, but do not establish clinical superiority over antibody-based checkpoint therapy.

An electrochemical assay for PD-L1-positive CTCs demonstrates reagentless monitoring in non-small-cell lung cancer [69]. It is relevant as a transferable analytical concept, but it cannot be cited as breast-cancer patient validation. Reviews of aptamer immunotherapy in TNBC and dual-checkpoint designs provide a broader context for immune engineering [70,71], while the disease-specific evidence remains predominantly preclinical.

#### 3.3.5. Stem-like and Stress-Associated Phenotypes

CD44 is associated with adhesion, plasticity, and stem-like states. A recent aptamer preferentially recognized chemoresistant mesenchymal stem-like TNBC cells and inhibited tumor growth in experimental models [72]. The truncated C24S sequence was reported to capture a high fraction of CD44-positive cells in spiked buffer, but patient-blood performance remains unestablished [73]. CD44/CD133-directed nanocarriers and CD44-aptamer liposomes demonstrate targeted formulation strategies [74,75], although receptor expression on normal and hematopoietic cells complicates specificity.

AS1411 is a 26-nt G-rich DNA aptamer that forms G-quadruplex structures and is commonly linked to cell-surface nucleolin. In breast-cancer cells, AS1411 was reported to destabilize BCL2 mRNA and promote cell death [76]. The precise mechanism remains debated because AS1411 activity depends on nucleolin localization, uptake pathways, and G-quadruplex-dependent interactions. Carbon-dot, gold-nanoparticle, chalcone, and controlled-release constructs extend AS1411 into detection, radiosensitization, and drug delivery [77,78,79,80]. Importantly, a Phase II study in metastatic renal-cell carcinoma—not breast cancer—reported acceptable tolerability but limited activity in an unselected population [81]. The trial demonstrates clinical exposure to AS1411; it does not establish a validation level of “breast-cancer clinical trial.”

#### 3.3.6. Multiplex Interpretation

Individual TNBC cells can express shifting combinations of EpCAM, CD44, integrins, PD-L1, Trop-2, EGFR and other markers, reflecting the molecular heterogeneity of TNBC [56,57]. Multicolor fluorescence, SERS barcodes, and heterovalent DNA frameworks can therefore recover information lost by single-marker averages [51,82,83]. A dual-aptamer ratiometric SERS platform measured breast-cancer extracellular-vesicle markers and illustrates the multiplexing capacity of distinct Raman reporters [82]. Yet multiplexing is not automatically clinically informative. A panel must define its denominator, measurement error, and decision rule, and the resulting cellular fractions must be associated prospectively with treatment response. The near-term value of TNBC aptamers lies in phenotype resolution, multiplex capture, and modular targeting rather than assignment of a new clinical subtype from a single research model. Their ability to combine multiple recognition elements may help accommodate the marked heterogeneity of TNBC. Representative subtype-associated aptamers and their validation status are summarized in Table 2. For Table 2, primary studies were considered when the breast-cancer-associated aptamer, molecular or cellular target, aptamer chemistry, selection strategy, binding characteristics, and principal functional application could be verified from the original publication.

**Table 2:** Representative subtype-associated breast cancer aptamers: molecular targets, binding affinity, and validation status.

Aptamer	Chemistry	Molecular Target (Subtype)	Selection Strategy	Binding Affinity	Principal Application	Critical Appraisal	Reference
HB5	ssDNA	HER2 peptide (HER2+)	Protein-SELEX (peptide)	Kd ≈ 18.9 nM (peptide); ≈316 nM (ECD)	ApDC; biosensor; serum probe	Widely reused; 17-fold affinity loss for ECD; no structural validation	[18,35,41]
* ERaptD4	ssDNA	ERα (Luminal)	HTS-assisted SELEX	Kd ≈ 6.45 nM	ERα detection in tissue/ELISA	κ = 0.930 (strong pos/neg, *n* = 30); κ = 0.823 (weak/moderate pos, *n* = 20); multicenter validation pending	[17]
MF3Ec	ssDNA	PHB2 (Luminal A)	Cell-SELEX (MCF-7)	Kd ≈ 18.95 nM	Luminal-associated tissue staining; imaging	Luminal-associated recognition, overlap in luminal B/HER2-negative tissue; tumor membrane PHB2 expression remains to be established.	[24,30,52]
SYL3C	ssDNA	EpCAM (broad)	Protein-SELEX	Kd ≈ 38 nM	CTC capture; NIR-II imaging	Workhorse for liquid biopsy; MDA-MB-231 is not an EpCAM-high model	[58,59,61]
AS1411	G-rich ssDNA	Nucleolin (TNBC)	Empirical discovery	No single comparable Kd assigned	Anti-TNBC agent; ApDC; imaging	Phase II trial in renal cell carcinoma; no breast-cancer clinical evidence; mechanism debated	[76,77,78,79,80,81]
Apt1/Apt2	ssDNA	MDA-MB-231 surface (TNBC)	Cell-SELEX + HTS	Kd 44.3 nM/17.7 nM	TNBC discrimination	HTS-enabled; targets not deconvoluted	[23]
PDGC21T	ssDNA	CD49c/integrin α3 (TNBC)	Cell-SELEX	Not quantitatively reported in the cited study	Cell recognition; target identification; xenograft imaging	Target deconvoluted; therapeutic translation pending	[31]
GreenB1	ssDNA	β1-integrin (TNBC)	Cell-SELEX	Kd ≈ 7 nM (FP)	Target identification and cellular internalization	Head-to-head clinical validation not performed	[32]

* For ERaptD4, the original study reported Ka = (1.55 ± 0.298) × 10^8^ M^−1^. The corresponding value of approximately 6.45 nM is the reciprocal of the reported point estimate under the same equilibrium model and should not be interpreted as an independently measured Kd [17]. Binding values obtained with peptides, recombinant proteins, and intact cells retain their original assay context [18,23,32]. A single Kd is not assigned to AS1411 because reported values depend strongly on target presentation, assay format, uptake pathway, and the still-debated mechanistic context [76,77,78,79,80,81].

## 4. Signal-Conversion Architectures: Mechanism Before Platform Labels

**Figure 4:**
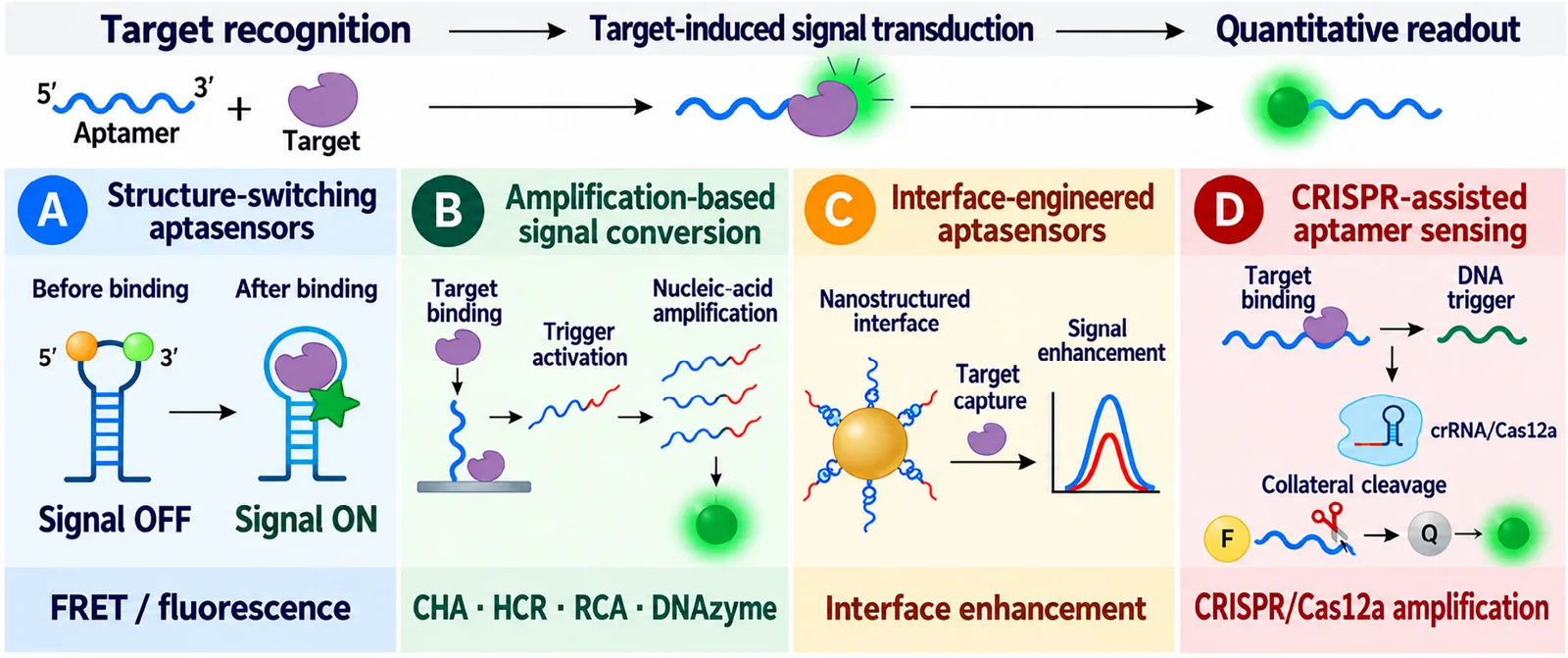
Mechanisms of signal conversion in aptamer-based biosensors. (**A**) Structure-switching aptasensors. (**B**) Amplification-based signal conversion through programmable nucleic acid circuits. (**C**) Interface-engineered aptasensors using nanomaterials for signal enhancement. (**D**) CRISPR-assisted aptamer sensing based on Cas-mediated signal amplification.

Although aptasensors are often classified by material or readout, their operation can be understood through several recurring signal-conversion mechanisms (Figure 4).

### 4.1. A Common Performance Framework

An aptasensor combines at least three functions: molecular recognition, conversion of binding into a measurable signal, and discrimination of that signal from background. LOD is consequently a system property. It depends on aptamer affinity and orientation, target presentation, surface density, nonspecific adsorption, amplification, calibration model and matrix [19,27]. Cross-platform ranking is particularly misleading when one assay measures purified HER2 in picograms per milliliter, another counts EpCAM-positive cells and a third measures vesicles per microliter.

Table 3 therefore reports target class, matrix, amplification, and clinical-sample status alongside the relevant performance metric. A result in buffer is not labeled “clinical” because a small number of patient sera was used for recovery, and a spiked-cell experiment is not treated as patient CTC capture. For every architecture, the relevant questions are: What initiates the signal? Which step amplifies it? Was the calibration range biologically plausible? How was LOD calculated? Were recovery, interference and between-batch reproducibility evaluated? Was the assay compared with a reference method in an independent cohort?

### 4.2. Binding-Induced Conformational Sensing

In a structure-switching sensor, target binding changes the distance between a reporter and a surface or separates a fluorophore from a quencher [8,27]. Methylene-blue- labeled electrochemical aptamers can translate this movement into a change in electron-transfer rate, while FRET pairs provide an optical counterpart. Such designs can be reagentless and compatible with repeated measurements, but only if the selected aptamer undergoes a sufficiently large and reproducible conformational change. Many aptamers bind through subtle local rearrangements, and immobilization can restrict the conformational ensemble.

HER2 electrodes based on carbon-nanotube affinity displacement, gold-nanoparticle-modified screen-printed carbon, or other conductive interfaces illustrate related surface-based formats [34,39]. ERα and CA15-3 systems similarly use surface-confined recognition [48,54]. Performance must be assessed in the intended ionic and protein environment: serum fouling and nonspecific changes in interfacial capacitance can mimic a binding signal. Reference electrodes, nonbinding sequence controls, and matrix-matched calibration are therefore mechanistic necessities rather than optional validation.

### 4.3. Amplification-Assisted Sensing

Hybridization chain reaction (HCR), catalytic hairpin assembly (CHA), rolling-circle amplification (RCA), enzyme catalysis, and polymer growth decouple target recognition from the magnitude of the final signal. Trop-2 recognition by aptamers was coupled with HCR for in situ cellular imaging and CHA for quantitative fluorescence amplification [66], whereas exosome detection platforms have employed dual RCA or aptamer-triggered tyramine amplification strategies [84,85]. A HER2 sensor coupled tetrahedral DNA and ARGET ATRP to generate a large electrochemical response from a small recognition event [36].

The gain in sensitivity comes with costs. Additional oligonucleotides increase leak reactions and contamination risk; enzymes require controlled storage; long incubation can undermine point-of-care claims; and each extra step creates a new source of lot-to-lot variability. Authors should report total assay time, hands-on steps, failure rate, and blank distribution, not only the final LOD. Amplification is valuable when it moves performance into a clinically necessary range, but unnecessary amplification can make a robust recognition event operationally fragile.

### 4.4. Nanomaterial-Enhanced Sensing

Nanomaterials increase surface area, control probe spacing, accelerate electron transfer, or concentrate optical fields. HER2 aptasensors have used enzyme-loaded covalent organic frameworks, MXene-like transition-metal materials, ZnO tetrapods, nickel foams, and gold composites [35,36,37,38,39]. MUC1 systems have used V2C/Au and three-dimensional CdCo nanosheets decorated with gold nanoparticles [86,87], while gold nanostars enable colorimetric cell detection [88]. These materials can produce strong signal-to-background ratios, but the quoted analytical gain includes the entire composite.

SERS provides narrow spectral fingerprints and high multiplex capacity. A dual-aptamer ratiometric SERS system used an internal standard to improve precision for breast-cancer extracellular vesicles [82], and an integrin α6β4-directed plasmonic assay was evaluated in a small plasma cohort [89]. The central manufacturing problem is hotspot reproducibility: small differences in nanoparticle size, aggregation, and surface chemistry can dominate Raman intensity [90,91]. Ratiometric designs, reference materials, and blinded between-batch testing are essential before clinical comparison.

Photoelectrochemical sensors separate optical excitation from electrical detection and can achieve low background, whereas field-effect transistor sensors offer direct electronic readout. FET biosensing in physiological media is constrained by Debye screening, which attenuates electrostatic signals from recognition events occurring beyond the electrical double layer [92]. Compact recognition elements such as aptamers, particularly when target binding induces charge redistribution close to the semiconductor surface, can partially circumvent this limitation [93]. Claims of direct detection in complex biological fluids should therefore specify ionic strength, sample pretreatment, and sensing-interface design.

### 4.5. CRISPR-Integrated Sensing

CRISPR-integrated aptasensors convert a protein- or vesicle-binding event into activation of a guide-dependent nuclease, after which Cas12a or Cas13 collateral cleavage amplifies a fluorescent, electrochemical, or chemiluminescent output. A 2024 chemiluminescence platform combined a CD63-based Cas12a assay for total exosomes with separate aptamer-chemiluminescence assays to estimate EpCAM- and MUC1-positive exosome fractions in plasma [94]. An electrochemical Cas12a aptasensor used magnetic capture for breast-cancer-derived exosomes [95].

The recognition target does not itself need a protospacer-adjacent motif; PAM dependence applies to the designed nucleic-acid activation step in particular Cas12a configurations [96]. It is therefore inaccurate to list “the protein target requires a PAM” as a generic limitation. More relevant constraints are the need to engineer a low-leak molecular bridge between aptamer binding and nuclease activation, the cost and storage of Cas reagents, and susceptibility of multistep reactions to contamination [96]. Clinical value will depend on whether collateral-cleavage amplification improves classification beyond a simpler aptamer assay.

### 4.6. Microfluidic and Paper-Based Point-of-Care Formats

Microfluidics integrates capture, washing, concentration, and analysis under controlled flow. Aptamer-cocktail nano-microfluidic chips and heterovalent DNA frameworks illustrate how multivalent geometry can improve capture of heterogeneous cells [83,97]. Pillar-based devices can profile CTC clusters through geometry and deformability even when epithelial state varies [98]. A PEGylated PDMS–titanium–gold device combined heterogeneous CTC capture with glutathione-controlled release and reported high post-release viability, enabling culture in the studied models [99].

These results should not be summarized as a universal 71–95% clinical capture efficiency. Capture depends on cell line, spike level, blood processing, flow rate, and marker density, and not all studies evaluated patient samples. Operational translation also requires control of clogging, channel-to-channel variation, sterility, and recovery losses. Paper and lateral-flow formats prioritize portability and simplified operation. MnO_2_-assisted dual-mode exosome detection and a dual-aptamer fluorescent vesicle assay show the potential for decentralized testing [100,101], but matrix robustness, shelf life, and reader standardization remain decisive.

Recent reviews of aptamer microfluidics summarize rapid technical diversification but reach the same conclusion: most platforms remain at proof-of-concept or small-cohort validation and cannot be compared without standardized sample processing [102,103].

### 4.7. Toward Minimum Information for Aptasensors

Systematic analyses of the aptamer literature have highlighted substantial inconsistencies in sequence reporting and validation, reinforcing the need for standardized reporting practices [104]. At minimum, an aptasensor report should identify the exact aptamer sequence and modification, target source and state, immobilization density, matrix and pretreatment, number and type of replicates, calibration model, blank distribution, LOD calculation, recovery, cross-reactivity, operator and batch reproducibility, total assay time, prespecified clinical comparator, and cohort design. This information is more useful for translation than another record-low LOD measured under incomparable conditions.

Existing reviews of analytical figures of merit for breast-cancer aptasensors provide a useful inventory, but future syntheses should prioritize evidence context and comparability over ranking isolated LOD values [19]. Quantitative performance metrics of representative aptasensor architectures are compiled in Table 3, with matrix and clinical-sample status explicitly distinguished. Despite these limitations, the modular integration of aptamers with electrochemical, optical, nucleic-acid amplification, and microfluidic platforms remains a major strength for developing multiplexed and adaptable sensing systems.For Table 3, representative aptasensor studies were selected to cover major signal-transduction architectures and were included when the target, analytical matrix, quantitative performance metric, and clinical-sample status could be verified from the primary report.

**Table 3:** Representative breast cancer aptasensor platforms: transduction mechanism, limit of detection, and clinical-sample context.

Target Category	Platform	Transduction & Amplification	Matrix	Performance Metric	Clinical-Sample Status	Key Limitation	Reference
Soluble HER2	COF-HRP electrochemical	Aptamer–antibody sandwich; HRP-catalyzed, APAP-mediated chronocoulometry.	Buffer calibration; serum spike recovery; separate clinical-serum analysis.	0.418 pg mL^−1^	Patient serum validation	Serum HER2 measurement does not establish tissue HER2 classification.	[35]
Soluble HER2	ZnO tetrapod-K4PTC	SWV; nanohybrid electrode	Spiked serum	0.58 fg mL^−1^	Spiked-serum evaluation	Calibration LOD does not establish clinical diagnostic sensitivity	[37]
Exosomes (EpCAM + MUC1)	CRISPR/Cas12a chemiluminescence	CD63-magnetic capture + Cas12a trans-cleavage	Exosome-concentration calibration; clinical plasma analysis	EpCAM: 1.45 × 10^2^; MUC1: 3.73 × 10^2^ particles µL^−1^	Clinical-plasma proof of concept	Prospective external validation needed	[94]
Exosomes (EpCAM/HER2)	Ratiometric SERS	Dual-aptamer displacement; rhodamine X/4-NTP Raman signal ratio	Cell-derived exosome calibration; clinical serum application	1.5 × 10^2^ particles mL^−1^	Clinical-serum application reported	Reproducibility of SERS hotspots unclear	[82]
CTC (EpCAM)	Liquid-crystal cytosensor	Optical/dielectric transduction	Cell-spiked human serum and whole blood	Optical: 5 cells; dielectric: 1 cell	Spiked-matrix evaluation	Patient performance not established	[59]
CTC (cocktail)	Microfluidic nanopillar chip	Multivalent capture	Artificial cell-containing samples; separate patient blood evaluation	71–83% capture in artificial samples	Patient-sample feasibility reported separately	Capture efficiency highly cell-line-dependent	[97]

Protein and vesicle concentrations, absolute cell numbers, and capture percentages describe different endpoints and are not directly comparable [35,59,94,97]. Calibration studies, spiked-matrix experiments, and measurements of endogenous analytes in patient specimens should be distinguished [35,37,82,97].

## 5. Liquid Biopsy: What Aptamers Can Capture and What They Cannot Read

**Figure 5:**
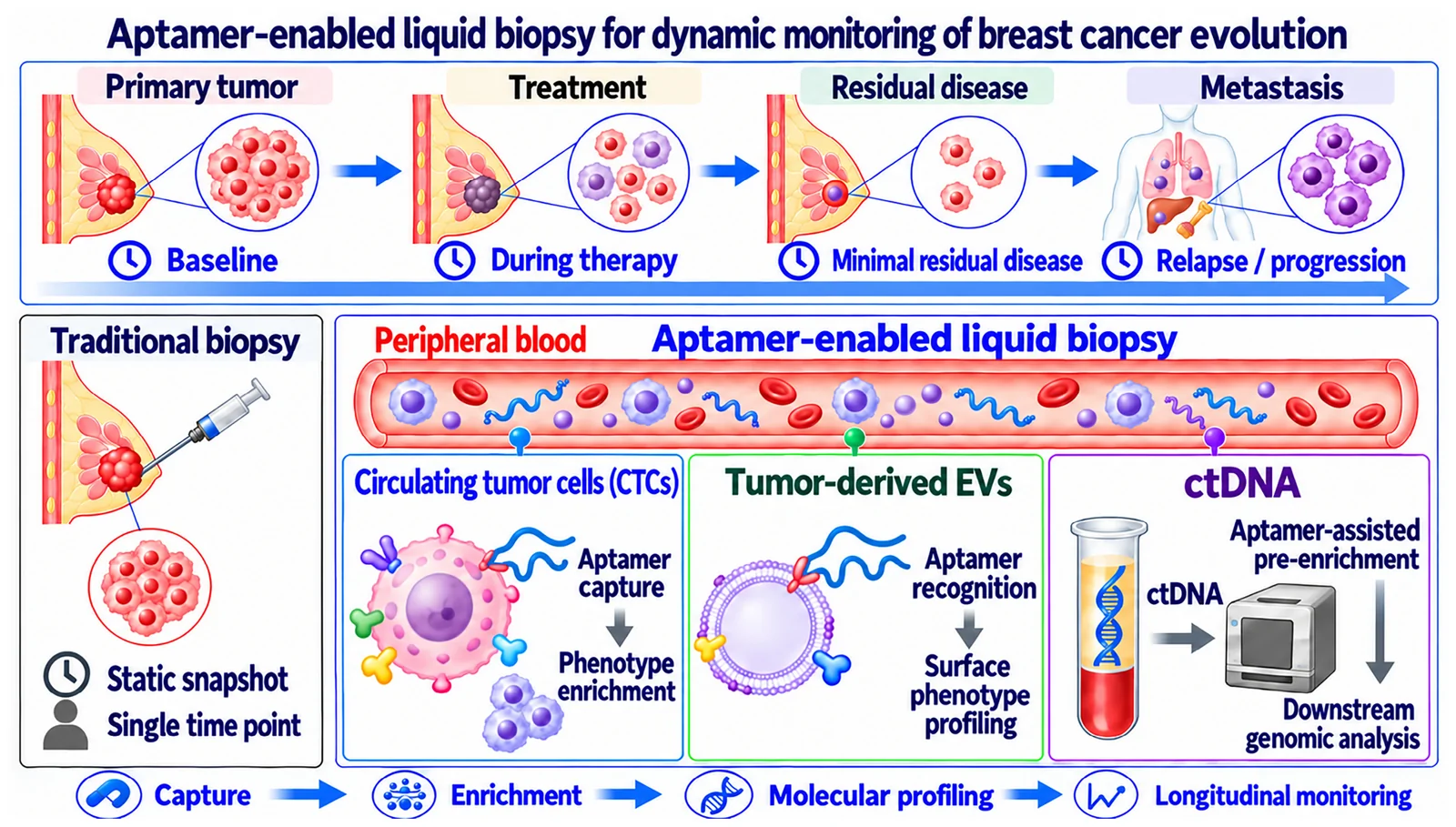
Aptamer-enabled liquid biopsy for dynamic monitoring of breast cancer evolution. Aptamer-based capture and enrichment of circulating tumor cells (CTCs) and tumor-associated extracellular vesicles (EVs) support molecular profiling and longitudinal monitoring across treatment and disease progression, complementing the static information obtained from conventional tissue biopsy. Plasma ctDNA analysis follows a separate extraction and sequence-specific detection workflow.

Aptamer-enabled liquid biopsy can provide repeated phenotypic information from CTCs and extracellular vesicles, complementing sequence-based ctDNA analysis during disease monitoring (Figure 5).

### 5.1. CTC Capture and Phenotyping

Liquid biopsy provides repeated access to tumor-associated material and may capture temporal evolution that a single tissue sample misses [105,106]. CTCs offer intact-cell phenotyping and potential downstream culture, but they are rare and biologically diverse. CellSearch is a clinically established prognostic CTC-enumeration system in metastatic breast cancer and relies on epithelial enrichment and cytokeratin-based identification [107]. Because CTC populations can include EpCAM-low or EpCAM-negative cells, EpCAM-only enrichment can undercount relevant populations.

Multimarker aptamer cocktails can be integrated onto microfluidic surfaces to improve capture of heterogeneous CTC phenotypes. Cocktail-functionalized chips and spatially ordered heterovalent frameworks improved capture across selected model cell lines [83,97]. However, the source studies do not constitute a direct, standardized, head-to-head demonstration that aptamer cocktails “surpass CellSearch.” Any comparison must use the same blood samples, pre-analytical workflow, reference definition, and clinically relevant endpoint.

Clinical feasibility of MDA-MB-231-aptamer-based CTC isolation was examined in breast-cancer patient blood samples, including preoperative and limited longitudinal follow-up sampling [108]. The study supported breast-cancer origin by orthogonal assays but found no significant association between CTC count and stage, subtype, metastasis, or progression; it therefore does not establish progression-free-survival utility or a validated longitudinal monitoring test [108]. Likewise, aptamer-targeted magnetic nanoparticles combined with microfluidics efficiently enriched CTCs in both experimental and patient samples [109]. Reversible release from aptamer-functionalized DNA frameworks can preserve cell integrity and viability for downstream analysis [83]. Those downstream applications remain prospective unless the complete workflow is validated from patient draw to actionable result.

### 5.2. Tumor-Associated Extracellular Vesicles

Extracellular vesicles are abundant components of liquid biopsy and carry surface proteins and internal nucleic acids, although tumor-derived vesicles coexist with vesicles released from non-malignant cells [105]. Their heterogeneous composition creates challenges for assigning tumor origin and requires additional molecular recognition strategies. Pairing vesicle-associated recognition with tumor-associated markers such as EpCAM, HER2, MUC1, or integrin-related signatures can improve the characterization of tumor-associated EV populations [82,89,110]. Click-chemistry fluorescence using a CD63 aptamer and polymer dots, aptamer-beacon-triggered surface-plasmon amplification and dual-RCA electrochemistry illustrate such strategies [84,85,111].

Exo-SELEX offers an alternative to assembling known markers. The 2′-fluoro-RNA aptamer ex-50.T was selected against breast-cancer-derived exosomes and reported to inhibit exosome uptake and exosome-induced migration in vitro [26]. Its molecular target remains undefined, and specificity across broader nonmalignant vesicle populations, particularly immune- and platelet-derived EVs, requires further investigation. Three questions remain central: can a platform attribute vesicle origin reproducibly, does analytical sensitivity yield clinically actionable specificity, and can longitudinal changes guide treatment rather than merely correlate with disease?

Ratiometric SERS detection of EpCAM/HER2-associated vesicles and integrin α6β4-focused plasma analysis show how multiplex or phenotype-specific measurements may improve discrimination [82,89]. The latter involved a small pilot cohort, so reported sensitivity and specificity should be treated as exploratory estimates. The Cas12a chemiluminescence study similarly provides clinical-sample proof of concept [94], but prospective external validation and a locked decision threshold are needed before diagnostic claims.

### 5.3. Aptamer Phenotyping Alongside ctDNA Analysis

Aptamers should not be framed as direct readers of ctDNA sequence variants. Aptamers recognize molecular shapes and surfaces; they do not read PIK3CA, ESR1, or other sequence variants by Watson–Crick complementarity. Mutation-specific detection is performed by PCR, digital PCR, sequencing, or another nucleic-acid hybridization method. Aptamer-functionalized platforms can nevertheless enrich CTCs for downstream molecular or genetic analysis [97,108,109]. Aptamer-based protein phenotyping may provide an orthogonal layer alongside ctDNA analysis, but it does not constitute direct mutation recognition.

Isothermal amplification coupled to aptamer recognition amplifies a signal generated by a protein or cell target; it should not be described as direct ctDNA mutation detection unless a sequence-specific recognition module is explicitly present [112,113]. Clinically validated cfDNA assays and breast-cancer ctDNA studies illustrate the different evidentiary standard: analytical validation of variant detection, defined allele fractions, and longitudinal association with disease burden or endocrine resistance [106,114,115,116,117]. Aptamers may contribute CTC/EV enrichment or an orthogonal protein-phenotyping layer, but these approaches complement rather than replace genomic analysis [97,106,118,119].

### 5.4. A Liquid-Biopsy Translation Scorecard

Four levels should be reported separately. Analytical validity includes recovery, purity, precision, interference, and stability. Clinical validity asks whether the measured signal distinguishes or tracks the intended state in an independent cohort. Clinical utility requires evidence that the result improves a decision or outcome. Operational feasibility includes blood volume, processing time, equipment, cost, and reproducibility across sites. Across the studies reviewed here, most breast-cancer aptamer liquid-biopsy systems remain at the analytical-validation or exploratory clinical-validation stage. Nevertheless, their capacity for repeated molecular capture and phenotypic profiling provides a promising complement to tissue biopsy and sequence-based liquid-biopsy assays. Aptamers are therefore best positioned as enrichment and phenotyping tools rather than replacements for established genomic assays.

## 6. Theranostics: Target Recognition Is Necessary but Not Sufficient

### 6.1. Design Variables and Comparison with ADCs

Aptamer theranostics connect recognition to imaging, drug delivery, gene regulation, protein degradation, or immune-cell redirection [120,121,122,123]. Their modularity permits defined attachment of a payload and, in principle, control of linker chemistry and stoichiometry. Therapeutic performance nevertheless requires successful progression from target engagement to modality-specific downstream events, including internalization, intracellular trafficking, payload release, or effector engagement. Failure at any step can erase the advantage of selective binding.

Compared with an IgG, an aptamer is smaller and more amenable to solid-phase synthesis and site-defined conjugation. These attributes can improve tissue diffusion or construct homogeneity. Conversely, rapid renal clearance can reduce exposure, and a monovalent ligand may lack the avidity and FcRn-mediated persistence of an antibody. ADCs benefit from established linker–payload engineering, validated pharmacology, and multiple regulatory precedents. No aptamer–drug conjugate (ApDC) has been approved for a solid tumor [124]. The proper comparison is therefore application-specific: can a particular aptamer construct produce a better therapeutic index, manufacturing profile, or controllable assembly than the relevant antibody platform?

### 6.2. ApDCs and Targeted Nanocarriers

HB5 was used to deliver cytotoxic cargo selectively to HER2-positive breast-cancer cells in vitro [18], and newer internalizing HER2 aptamers have been evaluated as theranostic or potential theranostic candidates [43]. AS1411 has been coupled to small molecules, combination chemotherapy, and targeted therapeutic applications [77,78,80]. CD44 aptamers have been displayed on liposomes or nanocarriers to target CD44-positive cancer-cell populations [74,75]. Aptamer-functionalized triptolide and other nanocarrier systems further illustrate targeted delivery strategies in breast-cancer models [121,125,126,127].

These studies often demonstrate preferential uptake or greater cytotoxicity in target-positive than control cells. Such contrasts validate recognition-dependent delivery but do not establish a therapeutic index in vivo. Mechanistic studies should therefore distinguish aptamer binding, internalization, cargo release, and payload action. Broader requirements for pharmacokinetics, biodistribution, safety, and comparator-controlled validation are considered in Section 7.

### 6.3. Endosomal Escape as the Central Intracellular Bottleneck

Receptor-mediated internalization does not guarantee productive delivery. Many nucleic-acid and macromolecular cargos remain in endosomes and are recycled or degraded in lysosomes, leaving only a small fraction available for cytosolic or nuclear action [128,129]. This bottleneck is especially important for siRNA, genome-regulatory cargos, and PROTACs, whereas a lysosomally released cytotoxic payload may exploit rather than avoid degradation.

pH-responsive, fusogenic, and membrane-active delivery strategies can increase endosomal release in broader delivery systems [130,131]. Evidence specific to breast-cancer ApDCs is limited, and gains in escape can increase membrane toxicity. For HER2 constructs, receptor downregulation, recycling, and lysosomal routing should be measured alongside uptake. Direct comparison with T-DM1 or T-DXd must consider that an ADC payload is designed around a defined intracellular release route [124]. Picomolar binding cannot compensate for a cargo that never reaches its intracellular target.

### 6.4. Gene Silencing and Targeted Protein Degradation

Aptamer–siRNA chimeras (AsiCs) integrate a targeting domain with an RNA-interference payload. A three-in-one construct aimed at the EGFR/HER2/HER3 network produced stronger activity than single-pathway approaches in reported HER2-positive models [44], while EpCAM-directed AsiCs knocked down survival or immune-regulatory genes in EpCAM-positive breast-cancer models [62]. The modular concept is strong, but target-mediated uptake, endosomal escape, and RNAi potency must be quantified separately to understand the source of efficacy.

Aptamer–PROTAC conjugates (APC) extend recognition to targeted protein degradation. The reported breast-cancer APC used an aptamer to improve tumor-selective delivery of a degrader [122]. PROTAC activity depends on intracellular concentration, formation of a productive target–PROTAC–E3 ligase ternary complex, and catalytic degradation kinetics [132,133]. Appending an aptamer can alter intracellular delivery and degrader accessibility, but whether this improves productive ternary-complex formation and protein degradation requires direct measurement. Comparative efficacy of APCs remains insufficiently defined.

### 6.5. Imaging and Phototherapy

Aptamers can carry fluorophores, radionuclides, magnetic materials, or phototherapeutic agents. An EpCAM-targeted SYL3C–PEG–ICG NIR-II probe improved intraoperative visualization and guided resection in a breast-cancer mouse model [61]. A glycoaptamer dual-target radionuclide construct enabled PET/CT imaging of TNBC models [134], and aptamer-assisted carbon-nanotube systems combined imaging with photothermal therapy [135]. Bispecific aptamer-decorated light-triggered nanoparticles targeted tumor and stromal components in breast-cancer-derived organoids, while other aptamer nanoformulations combined photodynamic and bioreductive treatment [136,137].

These platforms are preclinical. Modality-specific challenges include radiation or phototoxic dose, tissue penetration of excitation light, and demonstration that imaging information meaningfully guides intervention. “Theranostic” should describe a construct that genuinely links diagnostic information to intervention, not any particle carrying both an imaging label and a drug.

### 6.6. Immune-Cell Engineering and Modulation

Aptamers can be transiently displayed on immune-cell surfaces as targeting adaptors. Aptamer-engineered natural killer (ApEn-NK) cells were designed to enhance recognition and cytotoxicity against TNBC cells [123]. This non-genetic and transient surface engineering may offer greater controllability than permanent receptor engineering. The TNBC microenvironment can suppress NK-cell function through TGF-β and inhibitory checkpoint signaling, and clinical-grade NK products require controlled expansion, cryopreservation, and potency assays [138,139].

The PDGFRβ aptamer combination with anti-PD-L1 and the dual-function XQ-P3–paclitaxel construct show that aptamers can couple delivery with immune modulation [25,68]. Again, the appropriate conclusion is preclinical mechanistic potential. No evidence yet demonstrates improved patient outcome or a manufacturing advantage over established antibody or cell-therapy strategies. Although current evidence remains predominantly preclinical, the modularity of aptamers offers a distinctive opportunity to integrate molecular recognition with drug delivery, imaging, protein degradation, and immune-cell engineering within chemically defined constructs.

## 7. A Translational Roadmap

**Figure 6:**
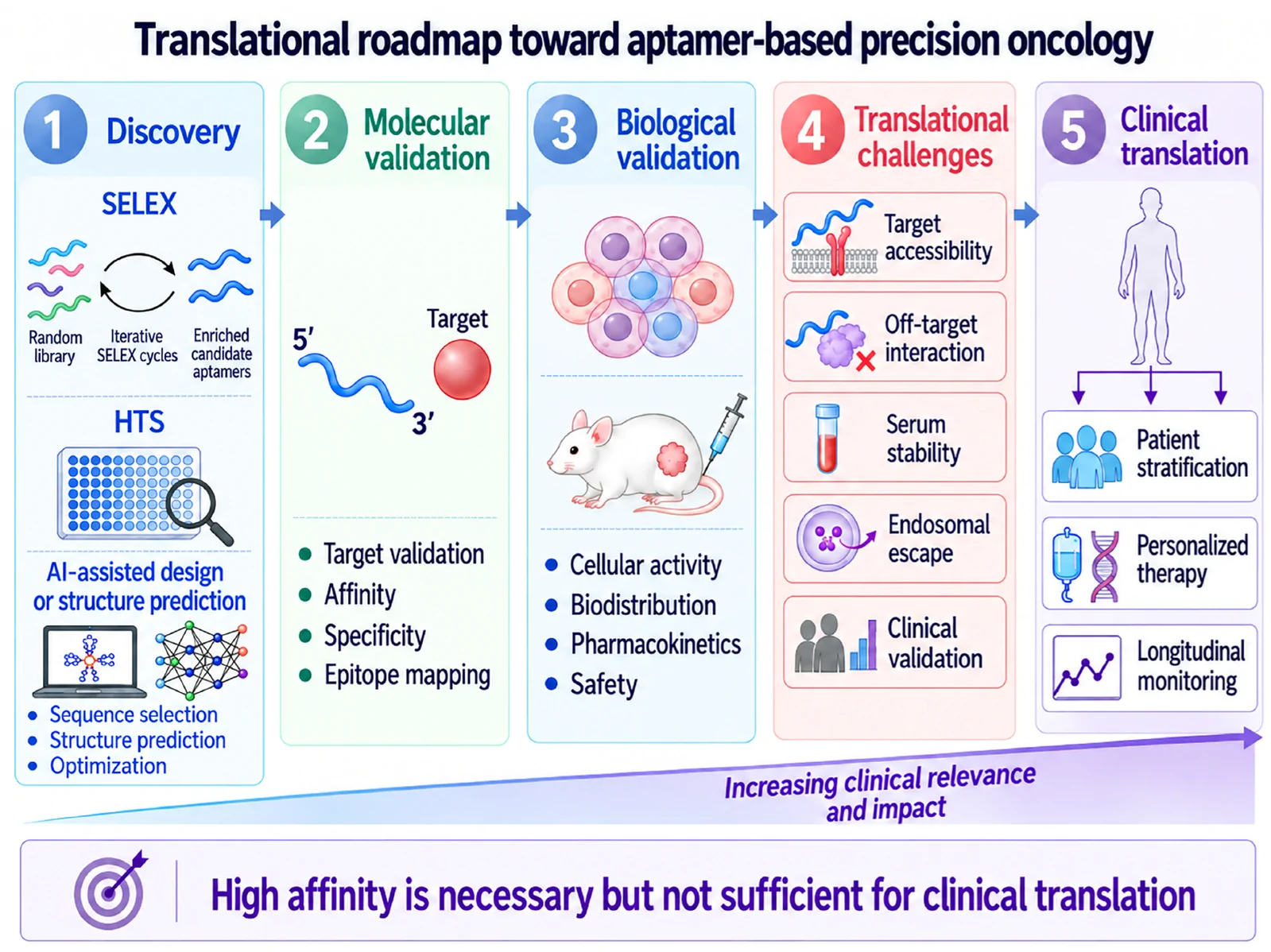
Translational roadmap for aptamer-based precision oncology. (**1**) Aptamer discovery and optimization. (**2**) Molecular validation of affinity, specificity, and target recognition. (**3**) Biological validation of activity, biodistribution, PK/PD, and safety. (**4**) Key translational challenges affecting clinical performance. (**5**) Precision medicine applications including patient stratification, personalized therapy, and longitudinal monitoring.

The modality-specific limitations discussed above converge on a common set of translational barriers. Progress toward clinical application therefore requires a staged framework integrating molecular validation, pharmacokinetics and biodistribution, safety assessment, analytical standardization, and comparator-controlled clinical studies (Figure 6).

### 7.1. Chemistry, Pharmacokinetics, and Biodistribution

The objective of chemical optimization is not maximal half-life in isolation but sufficient exposure at the biological site of action while preserving folding and specificity. For a surface sensor, stability during the assay may be enough. For systemic therapy, the sequence, terminal chemistry, conjugate size, plasma-protein interactions, and renal filtration jointly determine pharmacokinetics [7,8,9,140]. Every post-SELEX modification should therefore be accompanied by remeasurement of affinity, serum stability, and function, followed by biodistribution and toxicology appropriate to the final construct.

PEGylation can extend systemic exposure but may introduce immunological liabilities associated with pre-existing or induced anti-PEG antibodies [141,142]. Nanocarrier conjugation can likewise alter biodistribution and tissue access [143]. Quantitative PK/PD studies should relate intact construct exposure and released payload to target engagement [140,143].

### 7.2. Target Identity, Heterogeneity and Safety

Target deconvolution should precede therapeutic translation of a cell-selected aptamer [10,29]. A practical validation package includes orthogonal biochemical identification, loss-of-binding after genetic or pharmacological target perturbation, rescue when feasible, receptor-density measurement, internalization kinetics, and normal-tissue distribution. For an aptamer panel, each component needs the same characterization because nonspecific or redundant ligands can inflate apparent capture without improving phenotype resolution.

Breast-cancer heterogeneity makes multivalent or multiplex recognition necessary in many contexts, but it also complicates safety. CD44, integrins, nucleolin, and other “tumor-associated” molecules are not tumor-exclusive [144,145,146]. The relevant question is whether expression density, membrane organization, or internalization creates a sufficient therapeutic window. Patient-derived organoids, xenografts, and spatially resolved tissue panels can help define that window, but ultimately prospective clinical specimens are required [147,148].

### 7.3. Analytical Standardization and Staged Clinical Validation

Reproducibility begins with complete and accurate sequence reporting and transparent documentation of selection conditions [28,104]. Application studies should then lock the aptamer chemistry, surface preparation, calibration, and decision threshold before clinical evaluation. A staged program can prevent premature claims:

1. Discovery: Transparent selection, sequence disclosure, and orthogonal affinity measurement. 2. Biological validation: Target deconvolution, knockout or competition, internalization, and performance in relevant matrices. 3. Preclinical translation: Pharmacokinetics, biodistribution, toxicology, and comparator-controlled efficacy or analytical studies. 4. Clinical validation: A locked assay, prespecified endpoint, blinded multicenter cohort, and comparison with the clinical reference method.

For biosensors, authors should report analytical sensitivity, specificity, precision, interference, stability, and failure rate before clinical sensitivity and specificity. For CTC or vesicle systems, the event definition, denominator, and recovery standard must be explicit [149,150]. For therapeutic constructs, exposure, payload release, and target engagement should accompany tumor-volume curves. This hierarchy moves the field from attractive proof of concept to evidence that regulators and clinicians can interpret.

### 7.4. Structure-Guided and AI-Assisted Engineering

High-resolution aptamer–target structures could reveal contact networks, explain affinity loss between peptide and native protein, and guide modification [151]. HER2 is a clear priority because no aptamer–HER2 complex has been resolved. Chemical probing can provide experimental structural constraints, while computational three-dimensional modeling can complement these data [152,153].

Machine-learning approaches such as AptaNet predict aptamer–protein interactions from sequence-derived features, and generative models such as RaptGen explore sequence space conditioned on selection data [154,155]. AlphaFold and AlphaFold 3 have transformed protein structure prediction and expanded modeling of biomolecular interactions [156,157], but neither automatically validates a flexible aptamer complex. Alternative folds and conformational adaptation remain important limitations [151,153]. AI should therefore prioritize candidates and propose mutations within an experimentally closed loop [158].

Data quality remains a rate-limiting input: heterogeneity in selection conditions, affinity measurements, and sequence reporting can limit model transferability [28,104]. Standardized metadata and prospective, uncertainty-aware benchmarking are therefore essential for assessing generalizability [159].

### 7.5. Three Priorities for the Next Phase

First, structure-guided aptamer engineering should connect selection to experimentally constrained interfaces and systematic mutational maps. Second, multiplex molecular barcodes should quantify co-expression and cellular fractions, with prespecified links to therapy response rather than descriptive clustering alone. Third, clinically anchored AI-assisted discovery should optimize a composite objective—affinity, specificity, stability, internalization, and manufacturability—using standardized data. Across all three, the decisive advance will be a better validated molecule, not a more elaborate platform around an uncertain recognition element.

### 7.6. Implementation and Future Evaluation

Diagnostic development requires a defined specimen and patient population, reproducible processing, and comparison with an appropriate clinical reference [106]. CTC and EV studies also need clear event definitions and recovery controls [149,150]. Therapeutic constructs require evaluation of exposure, biodistribution, and payload-specific action [9,140]. We recommend suitable free-payload or carrier controls and evaluation of a fixed assay or formulation using prespecified endpoints and relevant comparators.

## 8. Conclusions

Aptamer technologies provide a programmable platform for breast-cancer molecular recognition, biosensing, liquid-biopsy enrichment, and targeted delivery. Their principal strengths lie in synthetic programmability, modular conjugation, reversible assembly, and compatibility with multiplexed recognition. However, affinity and analytical sensitivity alone do not determine translational value; selection context, target identity, matrix effects, pharmacokinetics, biodistribution, intracellular trafficking, and assay reproducibility are equally important. In liquid biopsy, aptamers are currently best positioned for molecular capture and phenotypic profiling rather than direct mutation detection, whereas therapeutic applications remain constrained by systemic exposure, internalization, productive cargo delivery, and safety.

The main contribution of this review is to connect subtype-associated aptamer recognition with the evidence required for a defined application. Rather than ranking platforms by affinity or limit of detection alone, we distinguish analytical performance, patient-level evidence, and clinical utility, and evaluate sensing, liquid-biopsy, and therapeutic systems using common translational criteria. Antibodies, IHC/ISH, genomic assays, and antibody–drug conjugates therefore remain essential comparators according to the intended task, while aptamers are most likely to provide added value where programmable chemistry, multiplex recognition, or controllable assembly offers a functional advantage.

Future work should prioritize orthogonal target validation, structure-guided optimization, standardized analytical reporting, multiplex phenotyping, and prospective studies using clinically relevant comparators. Diagnostic platforms will require locked assays, blinded evaluation, and independent cohorts, whereas therapeutic constructs will require quantitative pharmacokinetic, biodistribution, efficacy, and safety assessment. These priorities reflect the central proposition of this review: successful translation will depend not on increasingly elaborate platforms alone, but on demonstrating that a well-validated aptamer improves a defined measurement or intervention under biologically and clinically realistic conditions.

## Figures and Tables

**Table 1:** Scope and contribution of selected breast-cancer aptamer reviews and the present synthesis.

Review	Main Focus	Contribution	Scope and Limitations	Reference
Liu et al. 2017	Selection methods and breast-cancer diagnosis and therapy	Connects ligand discovery with application examples	Useful historical overview; predates the 2026 clinical-specimen studies discussed here	[20]
Ahirwar et al. 2021	Aptasensors for breast-cancer biomarkers and analytical figures of merit	Provides a basis for assessing sensing performance	Its stated focus is analytical sensing; comparison here concerns intended use across sensing and delivery	[19]
Chu et al. 2026	Aptamer properties, selection, diagnosis and treatment	Summarizes diagnostic platforms and therapeutic delivery, with a discussion of translational challenges	Organizes a broad application overview; the present review instead uses target identity, measurement context and clinical evidence as common comparison axes across applications	[21]
This review	Subtype-associated recognition across selection, sensing, liquid biopsy and delivery	Separates analytical performance, patient-level evidence and clinical utility	Critical narrative synthesis across heterogeneous study designs; not intended as an exhaustive systematic review	This work

## Data Availability

No new data were created or analyzed in this study. Data sharing is not applicable to this article.

## Supplementary Materials

The following supporting information can be downloaded at: https://www.mdpi.com/article/10.3390/bios16100549/s1, Table S1: Database-specific literature search strategies used during manuscript revision.
