# Supplementary material for: Subtype-Specific Aptamer Technologies for Breast Cancer: From Molecular Recognition to Precision Applications
Source: Biosensors (Basel). 2026 Oct 1;16(10):549. doi: 10.3390/bios16100549 (PMC13702483; doi:10.3390/bios16100549)
Supplement: Supplementary file 1 [file biosensors-16-00549-s001.zip › biosensors-4568737-supplementary.pdf]

Subtype-Specific Aptamer Technologies for Breast Cancer: From Molecular Recognition to Precision Applications

Huibin Wu [1,†], Zhi Zeng [1,†], Wenyi Huang [1], Xinyan Lin [1], Rui Tian [2], Jianmin Chen [1,*] and Qiulong Zhang [1,*]

1 Key Laboratory of Pharmaceutical Analysis and Medicine of Fujian Province, School of Pharmacy and Medical Technology, Putian University, Putian 351100, China; 13101430061@163.com (H.W.); zengzhi@ptu.edu.cn (Z.Z.); 15105942669@163.com (W.H.); nzy670@126.com (X.L.)

2 College of Biological and Food Engineering, Hubei MinZu University, Enshi 445000, China; 2021060@hbmzu.edu.cn

* Correspondence: jmchen@ptu.edu.cn (J.C.); zhangql@ptu.edu.cn (Q.Z.)

† These authors contributed equally to this work.

PubMed was used as the principal bibliographic database, and X-MOL Advanced Search was used as a supplementary literature-discovery platform. All searches were performed on 17 September 2026. The PubMed publication-date limit was 1 January 1990 to 17 September 2026; X-MOL was limited by publication year from 1990 through 2026.

Table S1. Database-specific literature search strategies used during manuscript revision.

Source	Search set	Exact search strategy	Records
PubMed	Core search	("Breast Neoplasms"[Mesh]<br>OR "breast cancer"[Title/Abstract]<br>OR "breast cancers"[Title/Abstract]<br>OR "breast carcinoma"[Title/Abstract]<br>OR "breast carcinomas"[Title/Abstract]<br>OR "breast tumor"[Title/Abstract]<br>OR "breast tumors"[Title/Abstract]<br>OR "breast tumour"[Title/Abstract]<br>OR "breast tumours"[Title/Abstract]<br>OR "mammary carcinoma"[Title/Abstract])<br>AND<br>("Aptamers, Nucleotide"[Mesh]<br>OR aptamer*[Title/Abstract]<br>OR aptasensor*[Title/Abstract]<br>OR SELEX[Title/Abstract]<br>OR "systematic evolution of ligands by exponential enrichment"[Title/Abstract])<br>AND<br>1990/01/01:2026/09/17[dp]	961
PubMed	Molecular subtype	("Breast Neoplasms"[Mesh]<br>OR "breast cancer"[Title/Abstract]<br>OR "breast carcinoma"[Title/Abstract])<br>AND<br>(aptamer*[Title/Abstract]	273

		OR aptasensor*[Title/Abstract]<br>OR SELEX[Title/Abstract])<br>AND<br>(HER2[Title/Abstract]<br>OR ERBB2[Title/Abstract]<br>OR "estrogen receptor"[Title/Abstract]<br>OR "hormone receptor"[Title/Abstract]<br>OR luminal[Title/Abstract]<br>OR "triple-negative breast cancer"[Title/Abstract]<br>OR TNBC[Title/Abstract])<br>AND<br>1990/01/01:2026/09/17[dp]	
PubMed	Biosensing	("Breast Neoplasms"[Mesh]<br>OR "breast cancer"[Title/Abstract]<br>OR "breast carcinoma"[Title/Abstract])<br>AND<br>(aptamer*[Title/Abstract]<br>OR aptasensor*[Title/Abstract])<br>AND<br>(biosensor*[Title/Abstract]<br>OR electrochemical[Title/Abstract]<br>OR fluorescence[Title/Abstract]<br>OR SERS[Title/Abstract]<br>OR CRISPR[Title/Abstract]<br>OR microfluidic*[Title/Abstract]<br>OR "lateral flow"[Title/Abstract]<br>OR colorimetric[Title/Abstract]<br>OR photoelectrochemical[Title/Abstract])<br>AND<br>1990/01/01:2026/09/17[dp]	381
PubMed	Liquid biopsy	("Breast Neoplasms"[Mesh]<br>OR "breast cancer"[Title/Abstract]<br>OR "breast carcinoma"[Title/Abstract])<br>AND<br>(aptamer*[Title/Abstract]<br>OR aptasensor*[Title/Abstract])<br>AND<br>("liquid biopsy"[Title/Abstract]<br>OR "circulating tumor cell"[Title/Abstract]<br>OR "circulating tumour cell"[Title/Abstract]<br>OR CTC[Title/Abstract]<br>OR exosome*[Title/Abstract]<br>OR "extracellular vesicle"[Title/Abstract]<br>OR "extracellular vesicles"[Title/Abstract]<br>OR ctDNA[Title/Abstract]<br>OR "circulating tumor DNA"[Title/Abstract])<br>AND<br>1990/01/01:2026/09/17[dp]	142

PubMed	Theranostic applications	("Breast Neoplasms"[Mesh]<br>OR "breast cancer"[Title/Abstract]<br>OR "breast carcinoma"[Title/Abstract])<br>AND<br>(aptamer*[Title/Abstract]<br>OR "aptamer-drug conjugate"[Title/Abstract])<br>AND<br>(theranostic*[Title/Abstract]<br>OR "drug delivery"[Title/Abstract]<br>OR "targeted delivery"[Title/Abstract]<br>OR imaging[Title/Abstract]<br>OR nanocarrier*[Title/Abstract]<br>OR nanoparticle*[Title/Abstract]<br>OR siRNA[Title/Abstract]<br>OR PROTAC[Title/Abstract]<br>OR photothermal[Title/Abstract]<br>OR photodynamic[Title/Abstract]<br>OR radionuclide[Title/Abstract]<br>OR "NK cell"[Title/Abstract])<br>AND<br>1990/01/01:2026/09/17[dp]	550

Table S1. Continued.

Source	Search set	Exact search strategy	Records
X-MOL	Core aptamer	Search scope: Title and Abstract<br>Keyword row 1: breast cancer<br>Connector: AND; keyword row 2: aptamer<br>Publication year: 1990 to 2026	1062
X-MOL	Core aptasensor	Search scope: Title and Abstract<br>Keyword row 1: breast cancer<br>Connector: AND; keyword row 2: aptasensor<br>Publication year: 1990 to 2026	155
X-MOL	Core SELEX	Search scope: Title and Abstract<br>Keyword row 1: breast cancer<br>Connector: AND; keyword row 2: SELEX<br>Publication year: 1990 to 2026	53
X-MOL	HER2 subtype	Search scope: Title and Abstract<br>Keyword row 1: breast cancer<br>Connector: AND; keyword row 2: aptamer<br>Connector: AND; keyword row 3: HER2<br>Publication year: 1990 to 2026	150
X-MOL	TNBC subtype	Search scope: Title and Abstract<br>Keyword row 1: breast cancer<br>Connector: AND; keyword row 2: aptamer<br>Connector: AND; keyword row 3: triple-negative breast cancer<br>Publication year: 1990 to 2026	122
X-MOL	Liquid biopsy	Search scope: Title and Abstract<br>Keyword row 1: breast cancer<br>Connector: AND; keyword row 2: aptamer	26

		Connector: AND; keyword row 3: liquid biopsy<br>Publication year: 1990 to 2026	
X-MOL	Drug delivery	Search scope: Title and Abstract<br>Keyword row 1: breast cancer<br>Connector: AND; keyword row 2: aptamer<br>Connector: AND; keyword row 3: drug delivery<br>Publication year: 1990 to 2026	163

* **Note 1.** The record counts shown are database or platform retrieval counts, not the number of included studies.

Note 2. The searches overlap substantially and their counts must not be added together.

Note 3. X-MOL constructs searches from separate keyword rows. The field entries and connectors shown in Table S1 reproduce the executed searches; PubMed field tags were not entered into X-MOL.

Note 4. X-MOL results included visibly duplicated records or versions. X-MOL was used for supplementary discovery, and bibliographic details were checked against primary publication records before citation.

Note 5. The strategy supports a structured narrative review and was not designed or reported as an exhaustive PRISMA-based systematic review.

Note 6. Additional target-specific searches and backward/forward citation tracking of key publications were performed where necessary to verify individual aptamers and primary studies; these supplementary searches were not treated as predefined search sets and their retrieval counts were therefore not reported separately.
